# RepTB: a gene ontology based drug repurposing approach for tuberculosis

**DOI:** 10.1186/s13321-018-0276-9

**Published:** 2018-05-21

**Authors:** Anurag Passi, Neeraj Kumar Rajput, David J. Wild, Anshu Bhardwaj

**Affiliations:** 1 Bioinformatics Centre, Institute of Microbial Technology, Council of Scientific and Industrial Research, Chandigarh, 160036 India; 2 Academy of Scientific and Innovative Research, Council of Scientific and Industrial Research, Training and Development Complex, CSIR Campus, CSIR Road, Taramani, Chennai, Tamil Nadu 600113 India; 3 School of Informatics, Computing, and Engineering, Indiana University, Bloomington, IN 47405 USA

**Keywords:** Tuberculosis, RepTB, Repurposing, Network based inference, Polypharmacology, MDR/XDR, Interaction

## Abstract

Tuberculosis (TB) is the world’s leading infectious killer with 1.8 million deaths in 2015 as reported by WHO. It is therefore imperative that alternate routes of identification of novel anti-TB compounds are explored given the time and costs involved in new drug discovery process. Towards this, we have developed *RepTB*. This is a unique drug repurposing approach for TB that uses molecular function correlations among known drug-target pairs to predict novel drug-target interactions. In this study, we have created a Gene Ontology based network containing 26,404 edges, 6630 drug and 4083 target nodes. The network, enriched with molecular function ontology, was analyzed using Network Based Inference (NBI). The association scores computed from NBI are used to identify novel drug-target interactions. These interactions are further evaluated based on a combined evidence approach for identification of potential drug repurposing candidates. In this approach, targets which have no known variation in clinical isolates, no human homologs, and are essential for Mtb’s survival and or virulence are prioritized. We analyzed predicted DTIs to identify target pairs whose predicted drugs may have synergistic bactericidal effect. From the list of predicted DTIs from RepTB, four TB targets, namely, FolP1 (Dihydropteroate synthase), Tmk (Thymidylate kinase), Dut (Deoxyuridine 5′-triphosphate nucleotidohydrolase) and MenB (1,4-dihydroxy-2-naphthoyl-CoA synthase) may be selected for further validation. In addition, we observed that in some cases there is significant chemical structure similarity between predicted and reported drugs of prioritized targets, lending credence to our approach. We also report new chemical space for prioritized targets that may be tested further. We believe that with increasing drug-target interaction dataset RepTB will be able to offer better predictive value and is amenable for identification of drug-repurposing candidates for other disease indications too.

**Electronic supplementary material:**

The online version of this article (10.1186/s13321-018-0276-9) contains supplementary material, which is available to authorized users.

## Background

Emergence of Multi-Drug Resistant (MDR) and Extensively-Drug Resistant (XDR) Tuberculosis is impeding the progress of combating the epidemic of Tuberculosis [1]. According to WHO, in 2015 1.8 million people died of TB and almost 480,000 diagnosed with MDR-TB globally [2]. It takes up to 2 years to cure MDR/XDR patients and more than 50% patients do not respond to the existing treatment regimens [3]. Additionally, the existing drugs in the TB regimen are toxic (Fluoroquinolones & Aminoglycosides show hepatotoxicity and renal toxicity, respectively) that deters compliance and leads to poor-treatment outcomes [4, 5]. Given that only 10% of the compounds go through from Phase I to final FDA approval [6] and high attrition rates of lead molecules passing from preclinical development to Phase I clinical studies [7], alternative strategies are needed. Drug repurposing is an attractive strategy to identify novel treatment options given that it may reduce R&D timelines by 3–5 years, have less development costs and the improved quality of success [8, 9]. The concept of drug repurposing is not new and many new drug indications have been identified serendipitously. The classic cases include Sildenafil that was initially approved for angina but repurposed for erectile dysfunction and Canakinumab which was initially tested for Rheumatoid arthritis but was repurposed to treat cryopyrin-associated periodic syndrome (CAPS) [10].

Palomino et al. [11] discussed the viability of drug repurposing methods in treatment of infectious diseases like Tuberculosis. Many of the drugs like Fluoroquinolones (Gatifloxacin and Moxifloxacin); rifamycins (Rifapentine and Clofazimine); oxazolinones (Linezolid and Sutezolid); and beta lactams (Meropenem and Clavulanate) have been repurposed against MDR/XDR TB [12]. Linezolid and Metronidazole have completed the Phase II while Gatifloxacin have completed phase III clinical trials [13]. More recently, cephalosporins in combination with Rifampicin and other anti-TB drugs like Bedaquline and Delamanid have also shown promising synergistic activity [14]. Besides the anti-infectives, non-anti-infective agents have also been repurposed against TB. These include: Entacapone and Tolcapone, that act as adjunct to treatment of Parkinson’s disease [15]; Thioridazine and chlorpromazine are drugs used in treatment of psychoses; and NSAIDs such as Diclofenac, Ibuprofen, and Carprofen also have anti-TB activity [16].

Efforts towards identification of new TB drugs followed either phenotypic screens or target based screens [17]. The first approach, relies on development of whole-cell screening assays and availability of library of chemical compounds against replicating and non-growing *Mycobacterium tuberculosis* (Mtb). The target based screening approach involves purification of target protein, further compound screening assays against the target and in vivo target validation. As opposed to phenotypic screening, the target-based drug discovery has been less successful with few examples like identification of drug-like inhibitors of EthR and malate synthase. In order to systematically explore the drug target space in TB, several computational methods have also been developed which have identified potential drug repurposing candidates for TB. Brindha et al. [18, 19] identified drug candidates against Mtb MurE and PknB using docking based virtual screening method. In addition, methods based on structural proteome of Mtb by Kinnings et al. [20] and identification of polypharmacological drugs by Ramakrishnan et al. [21] have also reported repurposing candidates against Mtb proteins. However, these methods of drug repurposing are limited by the availability of the 3D structure of the target and/or ligand. Consequently, network pharmacology based methods have been adopted to predict drug target interactions (DTIs), for example, pharmaco-chemical-genomics; Random Walk; gene expression and network based analysis, etc. [22–24]. More recently, Cheng et al. [25, 26] used Network Based Inference method to predict drug-target interactions but has not been applied to identify repurposing candidates for TB.

In this study, we have developed a drug-repurposing platform, ***RepTB***, using network pharmacology approach to identify potential repurposed candidates for TB. RepTB utilizes Gene Ontology (GO) based drug-target interaction (DTIs) network to compute association scores for identification of new DTIs. Once the DTIs are predicted, a combined evidence based approach is applied to shortlist the potential candidates. Furthermore, to assess the chemical diversity, a near neighbor analysis was performed using the chemical structure similarity index between the known and predicted chemical space. In the end, we propose synergistic DTIs that may be evaluated further as potential starting points in TB drug discovery pipeline.

## Results

1. Promiscuity in drug-target interactions from DrugBank

**Fig. 1:**
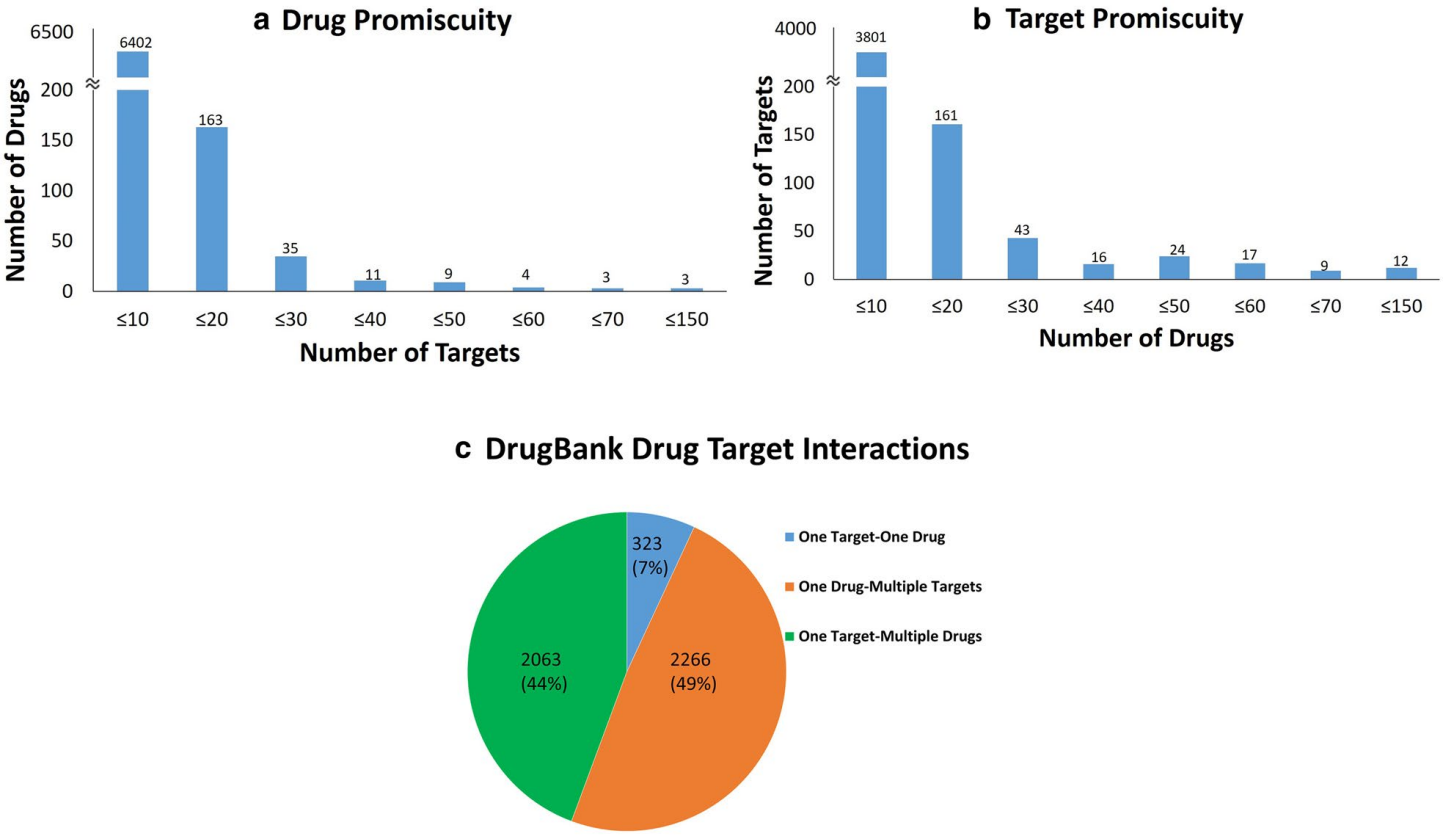
DrugBank data distribution. **a** The figure depicts the number of targets connected to each drug: shown as a frequency distribution graph in bins of 10. **b** Figure depicts the number of drugs connected to each target: shown as a frequency distribution graph in bins of 10. **c** The figure depicts the distribution of the DrugBank DTIs. The data clearly indicates that there is promiscuity in the drug-target interaction network that can be tapped to identify new interactions

A drug is promiscuous when it acts on multiple targets and exhibits distinct pharmacological effects [27]. To find out the level of promiscuity in the known drugs, we plotted the frequency of drugs against targets (Fig. 1). As seen in Fig. 1a, of the 6630 drugs from DrugBank [28–31], 6402 drugs bind to fewer than 10 targets of which 4364 drugs interact with only one target. The remaining 228 drugs interact with more than 11 targets, some of which are known to interact with wide range of targets (DB02379: 90 targets and DB00157: 144 targets). Likewise, of the total 4083 targets reported in DrugBank, 2020 targets bind to single drug (Fig. 1b). The rest, bind to more than one drug, for example, CDK2 protein (Uniprot ID: P24941) interacts with 114 drugs and Prothrombin (Uniprot ID: P00734) interacts with 101 drugs. In Fig. 1c, the DrugBank drug-target interaction network, 49% drugs and 44% targets show promiscuity. Of the total interactions, only 7% exhibit one drug-one target interaction (singletons).

**Fig. 2:**
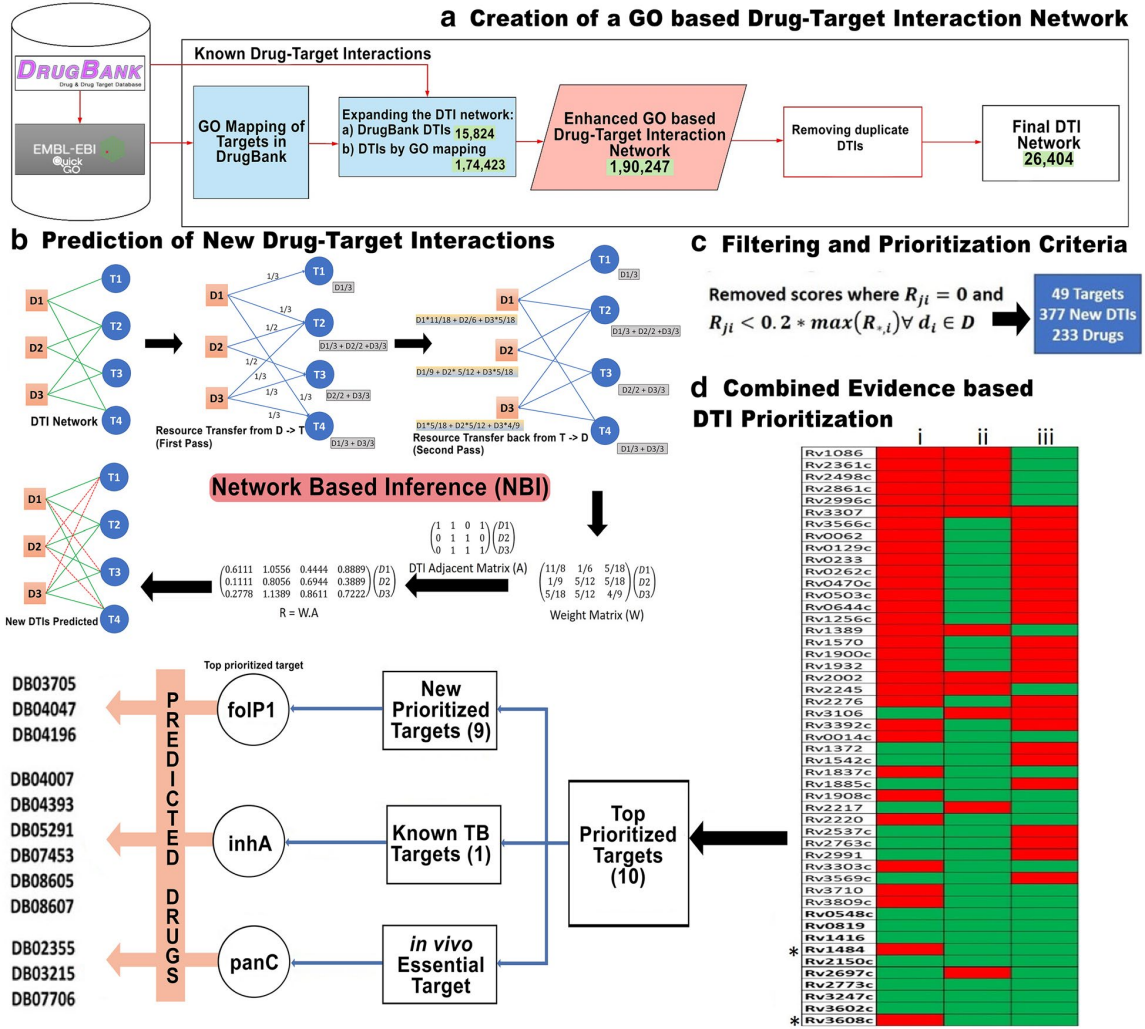
RepTB prediction workflow. **a** DrugBank DTI network was downloaded. Molecular function GO were mapped to the targets from DrugBank DTIs. Network was enriched by adding GO mapped DTIs to the network. The final network consists of 26,404 unique DTIs. **b** Network based inference (NBI) was used to predict new interactions between the drugs and targets (GO). Given a bipartite graph $G = \left( {N,E} \right)$ where $NisDT$(D is set of drug nodes, T is set of Target nodes), and E is edge between D and T. The green edges are the known DTIs and the red edges depict the predicted DTIs. A weight matrix is using NBI for the predicted and known DTIs. **c** Predicted edges were removed where predicted score $R_{ji}$ (where, R is the final resource matrix and *j* and *i* are the drugs and targets, respectively) was either zero or less than 20% of maximum DTI score for each drug. **d** 49 Mtb targets from DTI network were prioritized using combined evidence approach. A binary matrix was created with green (true) and red (false) placed for 4 conditions: (1) If syn/nonsyn variations are not present in the GMTV database. (2) If a human homolog is absent. (3) If the target is a reported essential gene. *Represents the target is present in prioritized list of targets from study done by Ramakrishnan et al. Representatives from the top 10 prioritized targets are shown—panC is essential in vivo, inhA is a known TB target. DrugBank Ids of the predicted drugs for the targets are also shown

As discussed above, the promiscuity in the drug-target interaction space may be harnessed to predict novel interactions for drug repurposing. For the same, a unique pipeline, RepTB is established which begins with creation of the Gene Ontology based network followed by a combined evidence based strategy to predict drug repurposing candidates . The pipeline is depicted in Fig. 2. The components of this platform are now discussed.

2. Gene ontology based enriched drug-target interaction network

The DrugBank DT network comprised of 15,824 edges. An additional set of 12,853 edges were added to the network based on molecular function Gene Ontology (GO) based mapping of the targets. On combining the DrugBank DTIs and GO mapping DTIs, the *RepTB* network contained 26,404 edges with 6630 drug and 4083 target nodes. The combined DTI edge list is available as Additional file 1.

3. Identification of new drug-target interactions using NBI

For the entire network comprising of 6630 drugs and 4083 targets, NBI association scores were computed for approximately 27 million (27,070,290) interactions. Of these, 26,404 are known and 27,043,886 are new associations (unconnected drug-target pairs). In order to create a prioritization list, we applied a metric where a target was only prioritized for a drug if its association score was more than 20% of the maximum score in the sorted list with unconnected targets. This lead to identification of 25,323 potential DTIs which are prioritized based on their NBI association scores.

4. Target prioritization from combined evidence approach

In the prioritized dataset of 25,323 DTIs, there are 49 Mtb targets interacting with 233 drugs (Additional file 2). In order to further prioritize targets from the potential DTIs, a combined evidence approach was implemented with three major criteria—role in drug resistance, absence of a human homolog and essentiality for survival or virulence.

Based on these criteria, of the 49 targets, 10 targets were prioritized. Of these 10, seven targets qualify for all three parameters. The remaining three were included in the prioritized list based on various factors. For example, targets, namely, FolP1 (Rv3608c) and InhA (Rv1484), were also prioritized by another group [21]. Mtb dUTPASE (Dut, Rv2697) is also prioritized despite that it shares a 39% sequence identity with the human dUTPase. One might argue that Dut might not be a viable target after all. However, according to the studies by Chan et al. [34] structural differences in the active site between Human and Mtb Dut enables development of inhibitors specific to Mtb. We do stress that it is imperative to understand the key structural features of various proteins to consider them as viable targets.

5. Repurposed drug candidates for TB

As depicted in Table 1, we predicted 57 potential drug candidates for the 10 prioritized targets. These targets belong to metabolic pathways that are essential for survival of the bacteria such as folate biosynthesis, pyrimidine biosynthesis, cell division, mycothiol biosynthesis, menaquinone biosynthesis, mycolic acid biosynthesis, lysine and riboflavin biosynthesis, and pentothenate biosynthesis. Although many of the predicted drugs are antibacterial, some drugs have reported indications as antiviral, antifungal, anti-cancer and treatment of bipolar disorder. Moreover, some nutraceuticals and obesity drugs are also predicted as potential candidates for TB targets.

**Table 1:** The table shows the predicted drugs and their indications for the top 10 prioritized targets

Target/GeneID	Pathway	Known drug	Predicted drug	Predicted drug indication
folP1/Rv3608c	Tetrahydrofolate biosynthesis	DB03592	DB03705;DB04047;DB04196	Antibacterial; antibacterial.; antibacterial
panC/Rv3602c	(R)-pantothenate biosynthesis	DB01930; DB02596;DB02694;DB03107	DB02355;DB03215;DB07706	Antibacterial; antibacterial; antibacterial
Tmk/Rv3247c	dTTP biosynthesis	DB01643;DB02452;DB03280;DB03666;DB03846;DB04160;DB04485	DB01799;DB02480;DB02594;DB02745;DB03150*;DB03165;DB03195*;DB03233*;DB03723;DB03845*;DB04170	Antibacterial; antibacterial; antibacterial; bipolar disorder; antiviral; antibacterial; anti-cancer; antibacterial; antibacterial; anti-cancer; antibacterial
dapB/Rv2773c	Lysine biosynthesis	DB04267	DB03969	Antibacterial
Dut/Rv2697c	dUMP biosynthesis	DB01965;DB02333;DB03413;DB03800	DB04685*	Antifungal
ftsZ/Rv2150c	Cell division	DB01864;DB04272;DB04315	DB00150;DB02082*;DB02547;DB02623*;DB02703;DB02975;DB03171;DB04124;DB04261;DB04723;DB06835;DB06921;DB07136;DB07157;DB07182;DB07269;DB08185	Nutraceutical; membrane transport inhibition; antibacterial; unknown; antibacterial; antibacterial; antibacterial; antibacterial; antibacterial; unknown; unknown; unknown; unknown; unknown; unknown; unknown; antibacterial
inhA/Rv1484	Mycolic acid biosynthesis	DB00609;DB00951;DB02990;DB04289;DB07090;DB07123;DB07155;DB07178;DB07188;DB07192;DB07222;DB07287;DB08604	DB04007;DB04393*;DB05291;DB07453;DB08605*;DB08607	Antibacterial; antibacterial; anesthetic; unknown; antibacterial; unknown
ribH/Rv1416	Riboflavin biosynthesis	DB01692;DB02135;DB02184;DB02290;DB02693;DB02711;DB03022;DB03812;DB03973;DB08016	DB02452	Antibacterial
mshD/Rv0819	Mycothiol biosynthesis	DB01992	DB01669;DB01764;DB01783;DB01846;DB01856;DB02516;DB03134;DB03230;DB03699;DB03905;DB03912	Antibacterial; antibacterial; obesity; antibacterial; unknown; antibacterial; unknown; antibacterial; unknown; unknown; antibacterial
menB/Rv0548c	Menaquinone biosynthesis	DB01992;DB03059	DB01669;DB01764;DB01783;DB01846;DB01856;DB02039;DB02516;DB03134;DB03230;DB03612;DB03699;DB03905;DB03912	Antibacterial; antibacterial; obesity; antibacterial; Unknown; antibacterial; antibacterial; unknown; antibacterial; mitochondrial beta oxidation; unknown; unknown; antibacterial

**Fig. 3:**
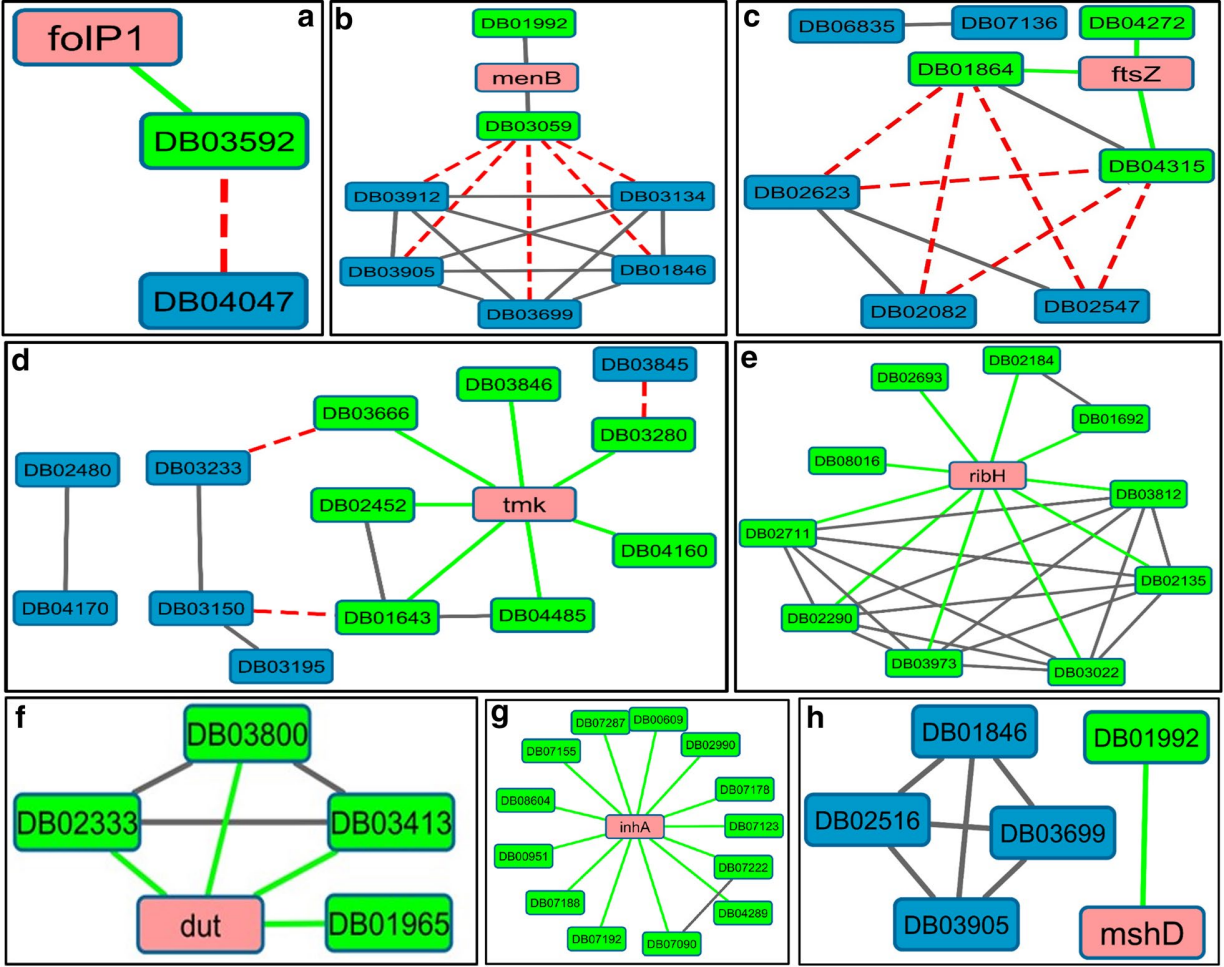
Predicted drugs for top 10 Mtb targets. The predicted targets are colored in pink. The known drug nodes of the targets are colored in green. The green edges show the known DTIs from the network. The dotted red line shows the highly similar (dissimilarity coefficient of 0.15) known and predicted drug for the specific target. Known and predicted drugs for FolP1, MenB, FtsZ and Tmk were observed to be structurally similar (panels a–d), no significant structural similarity was observed between the known and predicted drugs for Dut, InhA, RibH and MshD (panels e–h)

In order to understand the chemical diversity of the predicted drugs in context of the known drugs of the prioritized targets, chemical structural similarity analysis was performed. At a dissimilarity coefficient of 0.15, while known and predicted drugs for FolP1, MenB, FtsZ and Tmk were observed to be structurally similar (Fig. 3a–d), no significant structural similarity was observed between the known and predicted drugs for Dut, InhA, RibH and MshD (Fig. 3e–h).

## Discussion

*RepTB* is a systems based platform to identify repurposing candidates for TB. In this work, we have used a data driven approach towards prioritizing TB targets and identifying existing drugs that may act on Mtb. We use the concept of resource allocation between the connected nodes (reported drug-target pairs) to find association scores predicting the degree of association between previously unconnected drugs and targets. We use this concept to identify if a drug not known to be associated with a TB target can be repurposed for TB using NBI scores. Based on the combined evidence based approach, 10 Mtb targets were prioritized. These genes are deemed essential for Mtb and do not have human homolog. Recently, there has been a keen interest in identifying combinations of non-lethal genes whose incomplete inhibition can produce a lethal phenotype [35].

Rv3608c (FolP1, encoded as DHPS) is part of the folate biosynthesis in which the reduced folate species participate in one-carbon metabolism that produce biochemically important biomolecules such as purines, thymidine, methionine, serine, and N-formylmethionyl-tRNA. The dTMP formed as a by-product of folate biosynthesis feeds into the DNA synthesis. DHPS converts 2-Amino-4-hydroxy-6-hydromethyl-7,8-dihydropteridine-P_2_ to 7,8-Dihydro-pteroate which in turn converts to 7,8-Dihydrofolate (DHF) that gets converted into folate by dfrA. Mtb is unable to acquire folate from environment and is dependent on de-novo synthesis [36]. Dapsone and other sulfonamides have been known to inhibit folP1 by exhibiting bacteriostatic effect [37, 38]. Sulfa drugs target the folP encoded protein DHPS. Sulfa drugs are structural analogs pABA and act as competitive antagonists of DHPS. In addition, these drugs can be alternate substrates of DHPS and form sulfa-pteroates that cannot be further converted to folate that leads to a dead end in the pathway. Recently, mutations in the sulfa drug binding pockets of DHPS have caused resistance towards these drugs that has led to identification of alternate binding site in DHPS [39, 40]. The pterin binding pocket within the DHPS has a high degree of conservation and no sulfa drug resistance has been reported [41]. Hence, there have been studies designing compounds for inhibiting DHPS by binding to pterin binding pocket [42]. Three drugs were predicted for FolP1. DB04196, DB03705, and DB04047 are known to target *Bacillus anthracis* FolP protein and DB04047 is also known to target *B. anthracis* FolP and *E. coli* FolK proteins. The molecular function GO terms of *folP* and *folP1* genes are same (GO:0004156) even though the term assigned to *B. anthracis folP* gene is by IEA (Inferred from Electronic Annotation). Folk differs from FolP1 by transferring phosphorus-c moiety rather than alkyl or aryl group. These drugs can be repurposed against Mtb. The three predicted drugs for FolP1 are antibacterial drugs DB03705 (6-Methylamino-5-Nitroisocytosine), DB04196 (Pteroic Acid), and DB04047 ([Pterin-6-Yl Methanyl]-Phosphonophosphate). DB03705 and DB04196 interact with single targets in the DTI network while DB04047 interacts with only 2 targets in the network indicating low promiscuity in the DTI network suggesting a high specificity for the target. It has been reported that folP2 gene blocks sulfamethoxazole (SMX), which inhibits FolP1. This causes resistance against the drug. It was shown that combining FolP2 and FolP1 inhibitors can overcome the resistance in Mtb [38]. Similarly, in our study, the prioritized target FolP1 is similar to the known targets of its predicted drugs and those drugs do not act via binding to the sulfa binding sites. Hence, show no resistance.

Rv3247c (Tmk) and Rv2697c (Dut) are involved in Pyrimidine biosynthesis. Thymidylate kinase (Tmk) of Mtb is essential for DNA synthesis by converting dTMP to dTDP. This enzyme lies in the junction of de novo and salvage pathways for biosynthesis of dTTP [43]. It also utilizes dUMP as substrate however with low affinity than dTMP [44]. Out of 11 predicted drugs for Tmk, 7 are gram-negative antibacterial drugs namely, DB01799 (4-Hydroxy-3-Methyl Butyl Diphosphate), DB02480 ((S)-4-bromo-3-hydroxy-3-methylbutyl diphosphate), DB03165 (2-Dimethylamino-Ethyl-Diphosphate), DB03233 (Phosphoric Acid Mono-[3-Amino-5-(5-Methyl-2,4-Dioxo-3,4-Dihydro-2h-Pyrimidin-1-Yl)-Tetrahydro-Furan-2-Ylmethyl] Ester), DB04170 (4-bromo-3-hydroxy-3-methyl butyl diphosphate), DB02594 (2′-Deoxycytidine), DB03723 (2′-Deoxy-Thymidine-Beta-L-Rhamnose). One is an antiviral drug DB03150 (2′,3′-Dideoxythymidine-5′-Monophosphate), one is a bipolar disorder drug DB02745 (Uridine) and two drugs, DB03195 (Phosphoric Acid Mono-[3-Fluoro-5-(5-Methyl-2,4-Dioxo-3,4-Dihydro-2h-Pyrimidin-1-Yl)-Tetrahyro-Furan-2-Ylmethyl] Ester), and DB03845 (P1-(5′-Adenosyl)P5-(5′-(3′azido-3′-Deoxythymidyl))Pentaphosphate) are indicated as anti-cancer. DB02594 is a known human TK2 and deoxycytidine kinase inhibitor, and *E. coli* class B acid phosphatase. DB03233, DB03150, DB03195, DB03845 are already known to interact with human Tmk (P23919) proteins. All these proteins share the same GO annotation thymidylate kinase activity (GO:0004798). DB01799, DB02480, DB03150, DB03165, DB03195, DB03233, DB03845, and DB04170 interact with single targets while DB02594 interacts with 3 targets, DB02745 and DB03723 interacts with only 2 targets each. Another predicted target, dUTPase (Dut) is required for the optimal growth of Mtb [45] plays and important role in the de novo and salvage biosynthesis of dTTP [46]. It maintains the dUTP/dTTP levels low by cleaving the dUTP to form dUMP and consequently preventing the incorporation of uracil into DNA. In addition, Dut is also responsible for production of dUMP is a precursor for dTTP formation for DNA synthesis. If the *dut* gene is non-functional, the uracil content goes up causing its incorporation into the DNA and consequently cell death [46]. Moreover, dUTPase knockouts are shown to be lethal in *E.coli* and *S.cerevisiae* [47, 48]. Hence, the prioritized Dut can be a viable target for TB. Predicted drug for Dut, DB04685 is an antiparasitic drug interacts with only 2 targets, a human Dut protein and a *P. falciparum* Deoxyuridine 5′-triphosphate nucleotidohydrolase. Both proteins have the same molecular function GO term as that of the prioritized Mtb target for dUTP diphosphatase activity (GO:0004170). According to the work done by Kaur et al., it is possible to predict the vulnerability of a target in silico and to assess the bactericidality based on the NAD/NADH redox ratio [49]. Knockout of some of the genes can lead to increase in this ratio that differentiates whether the mutation is bacteriostatic or bactericidal. The ratio under normal growth is maintained within 0.5–2. If the ratio increases above 5, the cidality increases. Both *tmk* and *dut* were assessed to be bacteriostatic genes [50] and since these genes belong to the same pyrimidine biosynthesis pathway, have no human homologs and are essential for the survival of Mtb, can be targeted together to produce a synergistic bactericidal effect.

Rv0548c (MenB) is part of menaquinone biosynthesis which is essential in the electron transport chain and oxidative phosphorylation for the survival of Mtb. Menaquinone is synthesized by *menA*-*G* genes. MenB converts O-succinylbenzoyl-CoA to 1,4-dihydroxy-2-naphthoic acid (DHNA). Menaquinone has to be made continuously to maintain the membrane stability during growth [51, 52]. Since Mtb does not have ubiquinone like other bacterial species, it is dependent on menaquinone for its ATP production. Inhibiting MenB will prevent transfer of electrons in ETC and consequently inhibit the ATP production. Thus, a cascading effect will also decrease the activity of various other ATP-dependent efflux pumps. Our analysis predicted 13 drugs for MenB. Seven of these drugs—DB01669, DB01764, DB02516, DB03230, DB01846, DB02039, and DB3912 are antibacterials. DB01783 (Pantothenic acid), is used in treatment of testicular torsion, diabetic ulceration, wound healing, acne, obesity, diabetic peripheral polyneuropathy and DB03612 (3-Hydroxybutyryl-Coenzyme A) is an inhibitor of mitochondrial beta-oxidation. DB03699, DB01856, DB03134 and DB03905 drugs have been reported to target an unknown prokaryotic organism. However, the GO terms for the targets do not match with the predicted target. Since these drugs show low promiscuity, targeting MenB can therefore be a viable method of disrupting the formation of menaquinone and consequentially hindering bacterial growth [52].

Rv0819 (MshD) is an acetyltransferase and is a part of the mycothiol biosynthesis. 11 drugs were predicted for MshD. DB01669, DB01764 (Dalfopristin), DB03230, DB03912, DB01846, DB02516 are all antibacterials. DB01783 has been investigated for its hypolipidemic effects and as cholesterol lowering agent. Quinupristin-Dalfospristin is a known inhibitor of *Enterococcus faecalis* and is bacteriostatic against the bacteria. Also, when treated against *Mycobacterium marinum* was less active than clarithromycin [53]. However, when given in combination with other drugs like doxcycycline and ampicillin, the synergistic effect enhances the bactericidal activity [54]. DB03134, DB03699, DB03905 have been known to target unknown prokaryotic organisms.

Rv2150c (FtsZ) is part of the cell division process and standard anti-tubercular drugs are shown to be bacteriostatic towards FtsZ [49]. With the exception of DB08185, which is interacting with 27 different targets in the DTI, all the 17 predicted drugs for FtsZ have a very low promiscuity. Known targets for DB04723, Db06835, DB06921, DB07136, DB07157, DB07182, DB07269 share the molecular function GO term with FtsZ up to the hydrolase activity after which FtsZ branches out to further annotation downstream. Moreover, known targets of DB02082 and DB02623 share the same depth of GO annotation as FtsZ suggesting that the predicted drugs could be explored for repurposing against FtsZ.

3,4-Dihydroxy-2-butanone 4-phosphate of pentose phosphate pathway is converted to 6,7-Dimethyl-8- ribityllumazine by Rv1416 (RibH). Since Mtb is not capable of taking riboflavin from the environment and riboflavin biosynthesis is absent in humans, RibH makes a great target for anti-tb drug therapy. Predicted drug DB02452 is promiscuous and interacts with 10 targets in the DTI. However, according to the GO annotations, the known targets of DB02452 shared only single depth of the GO annotation as having a catalytic activity. Rv3602c (PanC) is part of the (R)-Pantothenate biosynthesis. A gene silencing study performed on *panC* reported that silencing *panC* in vitro was bacteriostatic in nature [55]. Predicted drugs for PanC, DB03255, DB03215, and DB07706, are antibacterial drugs and interact with only one target each hinting of high specificity. Rv2773c (DapB) is a part of lysine biosynthesis that feeds into peptidoglycan biosynthesis. Predicted drug for DapB, DB03969 is known to interact with *E. coli* DapB and can be repurposed for Mtb DapB.

Recently, there has been a renewed interest in Rv1484 (InhA) which is already a known anti-tb target. Mtb has a single copy of enoyl reductase making *inhA* an essential gene. However, known anti-tb drug, isoniazid is an inhibitor of InhA that acts via an indirect mechanism of action as a prodrug. It needs to be first activated by KatG. Since, there have been known mutations in KatG that cause resistance towards isoniazid, new direct inhibitors of InhA have recently been reported [56]. Predicted drugs for InhA in our study, DB04007, DB04393, DB08605 are antibacterials, DB05291 is an anesthetic, DB07453 and DB08607 have unknown indications. Known targets for DB07452, DB04047 and DB08608 share some functional GO based association with InhA. All these targets show oxidoreductase activity.

Polypharmcology is a preferred strategy to target more than one protein in Mtb. However, it is also important that there are no anti-targets or off-targets of the predicted drugs in the host. In the combined evidence approach, targets that have similarity with the host proteome are systematically eliminated from the prioritized list. So in essence, drugs with multiple targets in Mtb but no or few targets in host may be prioritized for further testing.

We identified similarity between the known and predicted drugs of the targets. FolP1, Tmk, FtsZ and MenB predicted drugs showed over 85% structural similarity (Tanimoto similarity) with their known drugs. The predicted drugs, were subjected to FAF drug filter [57] to predict their ADME/tox properties. We used the XLOGP3 method as the logP computation program with following parameters as true: PPIHitProfiler, Filter undesirable substructures moieties, Filter Pan Assay Interference Compounds (PAINS) Filter (A, B, C), Lilly MedChem Rules (regular). Out of the 56 predicted drugs, 4 drugs, namely, DB01764, DB07453, DB04196 (Pteroic Acid) and DB03969 passed all the FAF filters. Pteroic acid interacts with only one target in the DTI suggesting that the drug is not promiscuous. The drugs passed the FAF filter with desirable solubility and bioavailability suggesting that these compounds can be repurposed for the Mtb. The predicted compounds therefore, offer a new set of diverse chemical space that can be tested further for their anti-tb activity.

**Fig. 4:**
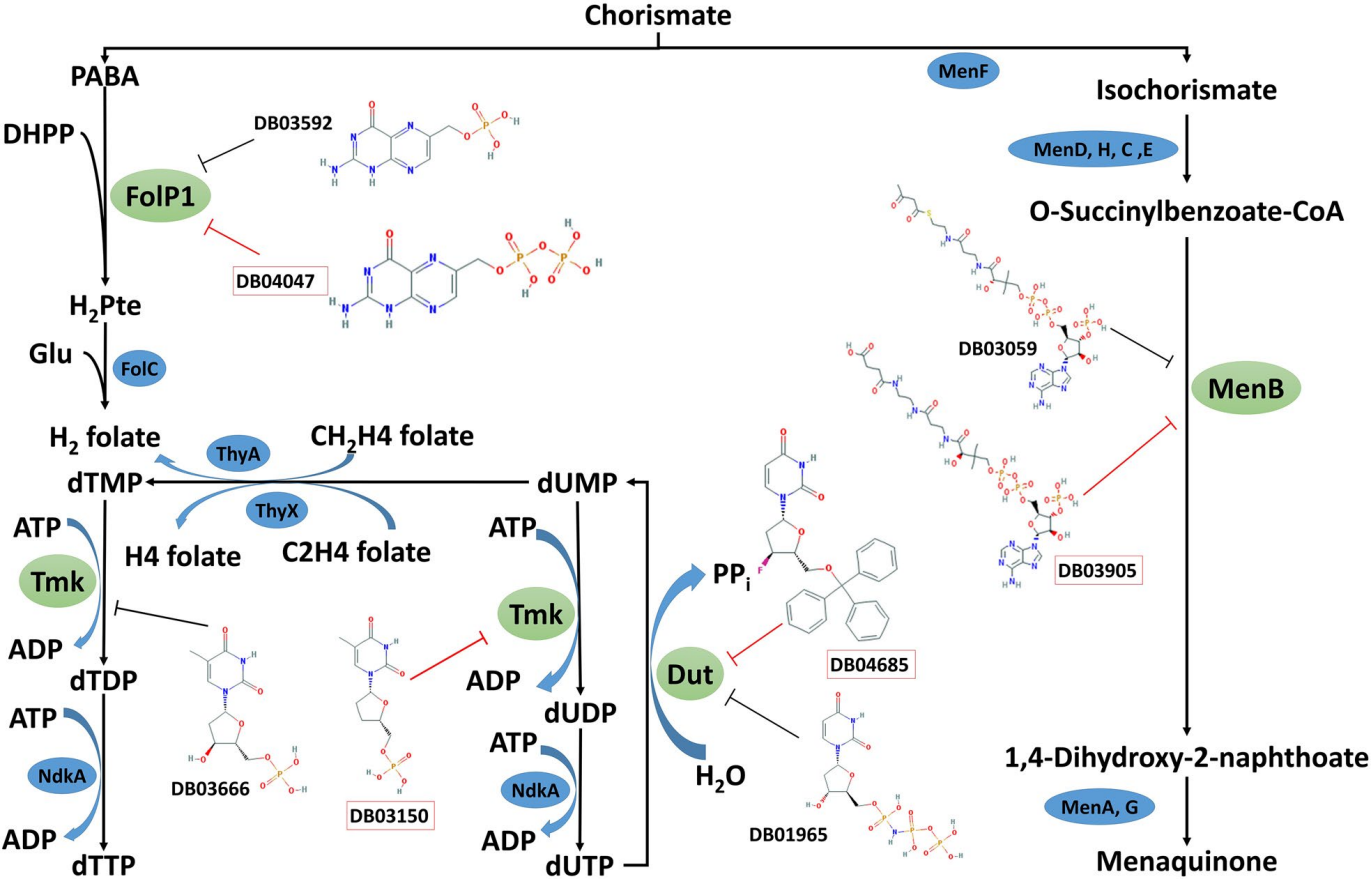
Proposed targets for synergistic inhibition. The targets are shown in green oval shape. The proteins in blue oval belong to pathways shown in the figure. The known drugs of these targets are in black and the predicted drugs for these targets are shown in red box

Based on the analysis done so far, it was observed that some targets of the predicted drugs fall in metabolic pathways that have common precursors and may be inhibited simultaneously for synergistic effect as shown in Fig. 4.

The structure based drug discovery process is based on the availability of three-dimensional structures of the target protein. Although there are 2052 Mtb structures in PDB, only ~ 700 representative structures for Mtb proteins are present. Therefore, alternate methods are needed to complement the structure-based prioritization of drug target space. For the predicted DTIs, we compared our results with those of other groups [20, 21] which are primarily based on the extent of structural information available for the Mtb proteins. Although there were no common predicted DTIs among the studies, there were common predicted targets such as InhA, PanC, MshD, FolP1, and MenB. Our work, therefore, complements published methods by providing new DTIs based on the molecular function associations between a drug and its known target. We have tried to incorporate GO information to the highest level of annotation for each target. Since, most proteins have broader GO terms, the functional aspect of DTI network is still a work in progress and as the GO terms for the targets become more descriptive, the GO mappings will be more precise leading to better predictions. We believe that RepTB may complement the efforts towards alternate process of drug discovery by predicting hidden drug-target associations.

## Conclusion

It is an established fact that TB is a global pandemic. There is an increasing demand for not only new drugs but also for innovative drug discovery strategies. In this work we created RepTB, a GO based DTI network to identify potential repurposed candidates for TB. RepTB uses combined evidence approach to prioritize Mtb targets. In addition, we also propose new chemical space that may be taken further for validation. Our method relies heavily on the availability of well-defined GO annotations and we believe that our method will be more accurate with improved annotations of the Mtb proteome. We, therefore, conclude that we have predicted diverse compounds that can potentially act as anti-tb. To the best of our knowledge, this is the first recommendation system to predict DTIs for TB through NBI and can also be applied for any other infectious agent.

## Methods

1. Data acquisition and network generation
  Known drug target interactions were taken from DrugBank [28–31] (version 4 downloaded in October 2015 and updated in February 2016). The original list contained 6630 unique drugs and 4083 unique targets. The total DrugBank DTIs were 15824. The data files, codes and associated documents are available through the GitHub link: http://ab-openlab.csir.res.in/gitlab/openlab/reptb/tree/master
2. Gene Ontology (GO) mapping for network enrichment
  We downloaded the molecular function GO mappings from QuickGO [32, 33] database of EBI. The data was filtered based on evidence codes mentioned in Additional file 3. Evidence codes IEA (Inference from Electronic Annotation), NAS (Non-traceable Author Statement) and ND (No Biological Data) were excluded as these three evidence codes either are not curated or do not have supporting reference. We mapped the leaf nodes of the GO annotations to DrugBank targets. If two targets shared the same GO code, a new DTI edge was created (http://ab-openlab.csir.res.in/gitlab/openlab/reptb/tree/master).
3. Drug-Target Network Projection using Network-Based Inference
  A drug-target interaction can be represented as a bipartite graph $G\left( {D,T,E} \right)$, where drug set $D = \left\{ {d_{1} ,d_{2} , \ldots ,d_{m} } \right\}$, target set $T = \left\{ {t_{1} ,t_{2} , \ldots ,t_{n} } \right\}$ and $E = e_{ij} :t_{i} T,d_{j} D$. An edge is formed if a drug is associated with a target. This graph can also be represented as an $n \times m$ adjacent matrix $\left\{ {a_{ij} } \right\}$, where $a_{ij} = 1$, if $t_{i}$ and $d_{j}$ are linked and $a_{ij} = 0$ if $t_{i}$ and $d_{j}$ are not linked.
  We denoted $f_{0} \left( o \right) = a_{io} ,o\left\{ {1,2, \ldots ,m} \right\}$ as the initial resource of drug $d_{o}$, for a target $t_{i}$, and $f\left( j \right)$ as the final resource of drug $d_{j}$. The final resource allocation can be depicted in a matrix form as $\mathop f\limits^{\prime }_{i} = W\mathop {f_{0i} }\limits^{\prime }$, where $\mathop {f_{0i} }\limits^{\prime }$ is the column vector of $f_{0}$ and $W$ is a weight matrix depicted as $W = \left\{ {w_{pq} } \right\}_{m \times m} = \left\{ {\frac{1}{{k\left( {tq} \right)}}\mathop \sum \nolimits_{l = 1}^{n} \frac{{a_{pl} a_{ql} }}{{k\left( {d_{l} } \right)}}} \right\}_{m \times m}$, where $k\left( {d_{l} } \right) = \mathop \sum \nolimits_{s = 1}^{m} a_{s} l$ represents the number of targets that interact with drug $d_{l} .$
4. Drug Target prioritization using recommendation system
  Predicted targets for each drug were prioritized by sorting in descending order of the NBI scores. DTIs with prediction scores of zero were removed and targets which had scores greater than 20% difference from the top score were taken as prioritized drug targets.
5. Prioritization of Mtb targets through combined evidence approach
  A target prioritization matrix was created using data from four different research studies to further prioritize Mtb targets. If a target from our study is also prioritized in any of the other studies, a “yes” is marked in that cell, otherwise a “no” is marked. The matrix also takes into account the essentiality of the gene, whether Nonsyn/Syn changes observed in clinical isolates, and whether a human homolog is present or not for that target. We performed a blastp sequence similarity search between the Mtb targets and the human proteome. The search parameters were based on e-value < 1E − 4,  % identity of < 35% and query coverage of >=60%. We also matched our set of prioritized targets witha study done by Ramakrishnan et al. [21] in which they predict DTIs by using sequence and structural analysis for understanding the evolutionary relationships between Mtb proteins and FDA approved drugs .
6. Near neighbor analysis to find chemically similar compounds
  We used ChemAxon’s JChem (Version 16.5.30.0) to find structurally similar compounds between known and predicted drugs for each of the target. Structural fingerprint for each of the drugs was calculated using the generatemd command from JChem as mentioned below:
**generatemd c < input_smiles_file > -g -k CF -f 1024 -D -o < output_filename>**
  Using this fingerprint, a tanimoto similarity coefficient was calculated to assess the structurally similar drugs using the nneib command:
**nneib -Xmx819 -f 1024 -t 0.3 -g < input_descriptor_file ≫ output_near_neighbor_file –v**

Cytoscape v3.5.1 [58] was used to represent the chemical space profiling for the above analyses.

## Additional files

**Additional file 1.** Combined DTI edge list.

**Additional file 2.** 49 Mtb targets with known and predicted DTIs along with components of combined evidence approach.

**Additional file 3.** GO Evidence Codes.
